# A review on selenium and gold nanoparticles combined photodynamic and photothermal prostate cancer tumors ablation

**DOI:** 10.1186/s11671-023-03936-z

**Published:** 2023-12-07

**Authors:** Olumakinde Charles Omiyale, Mariama Musa, Adewunmi Ifeoluwa Otuyalo, Tolulope Judah Gbayisomore, Damilola Zainab Onikeku, Solomon Damilare George, Possible Okikiola Popoola, Olabimpe Oluwatoyin Olofin, Kelechi Franklin Umunnam, Patricia Okwuchi Nneji, Musa Adnan, Taiwo Temitope Ogunjobi

**Affiliations:** 1 Department of Pharmacology, Toxicology, and Therapeutics, College of Medicine, University of Lagos, Lagos, Nigeria; 2 Department of Biomedical Engineering, University of Ghana, Legon, Ghana; 3 Department of Medicine and Surgery, College of Medicine, Ryazan State Medical University, Ryazan, Russia; 4 Department of Anatomy, University of Medical Sciences, Ondo, Nigeria; 5 Department of Health Promotion and Environmental Health Education, Faculty of Education, University of Ilorin, Ilorin, Nigeria; 6 Department of Genomic Research, Fresenius Medical Care, Lexington, USA; 7 Department of Physiology, Ladoke Akintola University of Technology, Ogbomoso, Nigeria; 8 Department of Biochemistry, School of Life Science, Federal University of Technology, Akure, Nigeria; 9 Department of Medical Laboratory Science, Vision College of Medical Sciences and Health Technology, Mbaise, Nigeria; 10 Department of Biology Education, Faculty of Science Education, Niger Delta University, Amassoma, Nigeria; 11 Department of Public Health and Healthcare, First Moscow State Medical University, Sechenov, Moscow, Russia; 12 Department of Biochemistry, Faculty of Basic Medical Sciences, Ladoke Akintola University of Technology, Ogbomoso, Nigeria

**Keywords:** Gold nanoparticles, Selenium nanoparticles, Photothermal therapy (PTT), Photodynamic therapy, Prostate cancer, Nanoparticle therapy, Polymer-based microcarriers, Reactive oxygen species (ROS)

## Abstract

The acceptance of nanoparticle technology in the quest for cancer treatment is due to its many potentials and possibilities of filling in the gaps in the limitations of the current treatment modalities. Insights into the possibilities of getting even more from this technology, as well as the synergistic properties of photothermal therapy (PTT) and photodynamic therapy (PDT)—the use of reactive oxygen species (ROS)—can also be exploited in the ablation of prostate cancer tumors. Therefore, the combination of gold and selenium photoactive nanoparticles as platforms for drug delivery via PTT/PDT in prostate cancer therapy, with a specific emphasis on the 'micro-carrier' based approach, was discussed and explored in this review under relevant subtopics ranging from understanding the complex chemistry and biology of the pharmacologically active Se/Au-containing agents to giving a thorough knowledge of these therapeutic agents' potential as a targeted and successful treatment strategy for prostate cancer by investigating the complex mechanisms behind their delivery, activation, and synergistic effects. Furthermore, this article presents a comprehensive overview of the current research environment, problems encountered, and future perspectives in the continuous war against prostate cancer.

## Introduction

Prostate cancer is a prevalent disease that constitutes a significant contributor to mortality rates associated with cancer among males on a global scale [1]. The condition typically originates from prostate cells and commonly manifests as either a localized or metastatic neoplasm. The significant morbidity and mortality associated with prostate cancer necessitate the exploration and implementation of novel therapeutic approaches. According to [2], The use of selenium and gold in delivery methods is a potentially advantageous strategy for addressing prostate cancer. The anticancer properties of selenium have been substantiated through its ability to modulate redox signaling pathways and facilitate apoptosis triggered by oxidative stress [2].

Gold nanoparticles have distinct photoactive characteristics that facilitate accurate tumor localization and localized hyperthermia [3]. The current landscape of cancer treatment using hyperthermia include extensive research on several techniques, including microwave stimulation, sound waves, photothermal therapy (PTT) using lasers, and photodynamic therapy (PDT). Nevertheless, like traditional cancer therapies, these treatment alternatives may lack specificity towards tumors and result in unintended consequences [4]. Therefore, there is a need to develop novel therapeutic approaches that can offer enhanced tumor control through synergistic benefits, minimized adverse effects, and increased practical applicability. In recent times, there has been a significant surge of interest in the field of multimodal combination of photoactive materials for cancer treatment, particularly the utilization of photothermal materials such as gold and selenium nanoparticles (NPs).

According to a study conducted by [5], there is currently no existing drug carrier that enables targeted heating specifically at the tumor site, which is a crucial requirement for the widespread application of photothermal therapy (PTT) in practical settings. The researchers conceived and developed a hybrid carrier with multilayer capsules. This carrier is incorporated with selenium nanoparticles (SeNPs) and gold nanorods (Au nanorods) to facilitate the generation of reactive oxygen species (ROS)-mediated photodynamic therapy (PDT) and photothermal therapy (PTT) multimodal combination. The findings from this and other studies, indicate that the use of selenium and gold nanoparticles within microcarriers holds significant promise for generating synergistic therapeutic outcomes in the treatment of prostate cancer [1, 3, 5].

The synergistic interplay of selenium nanoparticles (SeNPs) and gold nanoparticles (AuNPs), along with their straightforward production, cost-effectiveness contributes to their many advantages and their high surface-to-volume ratio, facilitates their easy infiltration into tumor sites via the enhanced permeation and retention (EPR) phenomenon. Selenium nanoparticles (SeNPs) can induce the generation of reactive oxygen species (ROS), which can function as an additional mechanism for suppressing tumor growth. The combined effect of these components boosts the effectiveness of thermal energy dissipation in the designed capsule. Consequently, this combined effect of SeNPs and AuNPs yields a dual pharmacological effect and enhanced photothermal conversion efficiency [1, 3, 5].

**Fig. 1:**
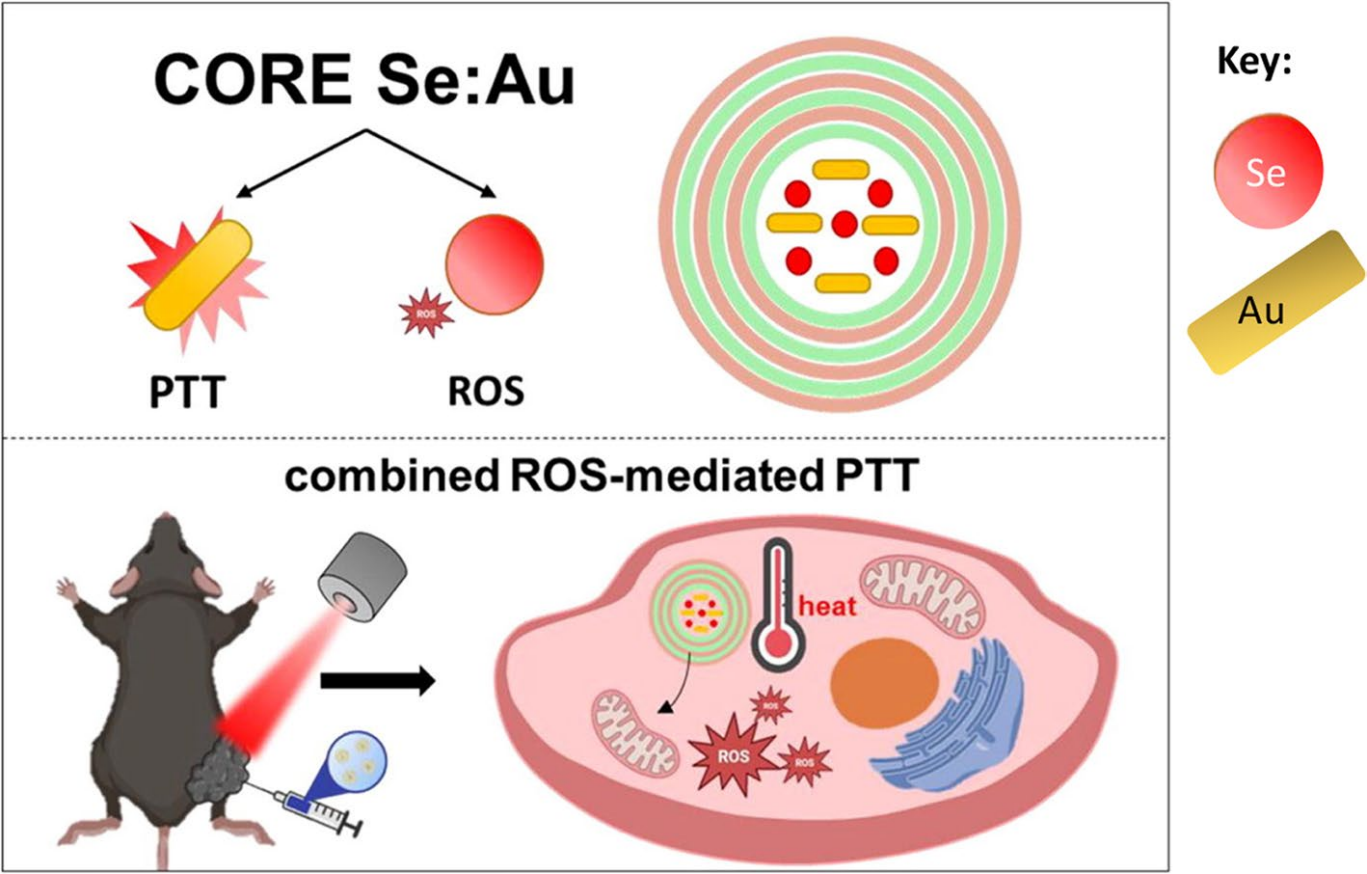
Overview of photo-active selenium and gold nanoparticles in PTT and the synergistic roles of ROS. This is adapted from [5]

The findings from both in vitro and in vivo experiments demonstrate that the combined use of photothermal treatment (PTT) and reactive oxygen species (ROS)-mediated therapy has a superior capacity to inhibit tumor growth compared to monotherapy approaches based on either selenium or gold-filled caps alone. In addition, the hybrid carriers have minimal in vivo toxicity towards vital organs like the heart, lungs, liver, kidneys, and spleen, as indicated by previous research [5]. The provided graphic illustrates a visual representation of the theoretical framework of this present study, referred to as Fig. 1 [5].

### Objectives of the literature review

The objective of this review article is to present a thorough overview of existing studies pertaining to the use of selenium and gold in delivery systems for the combined application of ROS-mediated photodynamic treatment (PDT) and photothermal therapy (PTT) in the context of prostate cancer. And, to provide a comprehensive understanding of the underlying principles of photothermal treatment, elucidate the role of reactive oxygen species (ROS) in cancer therapy, conduct a thorough examination of the mechanisms involved in nanoparticle transport, analyze the design and production of microcarriers, and assess their potential impact on enhancing therapeutic effectiveness. This paper aims to critically examine current advancements in microcarrier design, targeting methodologies, and therapeutic outcomes, with the objective of offering valuable perspectives on the potential of these emerging delivery systems to revolutionize the field of prostate cancer therapy.

#### Overview of the literature

A comprehensive understanding of the multimodal properties of selenium and gold-incorporated polymer-based microcarriers in the context of prostate cancer treatment is presented. Specifically, the article explores the potential of these therapeutic agents as a targeted and effective strategy for prostate cancer treatment by examining the intricate mechanisms involved in their delivery, activation, and synergistic effects in ROS-mediated combination photodynamic therapy (PDT) and photothermal therapy (PTT).

#### Scope and focus of the review

This review explores the intricate relationship between nanoparticle delivery pathways, tumor microenvironment, and therapeutic efficacy in prostate cancer cells with a specific emphasis on the 'micro-carrier' based approach. It highlights challenges and strategies for improving microcarrier design, focusing on recent papers published within the last 4 years.

### Methodology

This review summarizes publications pertaining to the subject matter. Keywords include "gold nanoparticles", "selenium nanoparticles", "photothermal therapy", "reactive oxygen species", and "photodynamic therapy". PubMed, Google Scholar, Web of Science, and Scopus were used to source quality peer-reviewed articles from 2018 to mid-2023. Clinical study data was obtained from the official website clinicaltrials.gov. This study holds significance as it highlights relevant research on the use of AuNP/SeNP microcarriers for the purpose of photothermal and ROS-mediated combination therapy in the context of prostate cancer. The objective of this study is to provide a comprehensive overview of recent advancements in the field of chemical modification, architectural design, and latest innovations of gold nanoparticles (AuNPs) and selenium nanoparticles (SeNPs) as microcarriers for reactive oxygen species (ROS)-mediated combination therapy targeting prostate cancer tumors.

## Overview of prostate cancer

### Epidemiology and clinical significance

Prostate cancer is widely recognized as the second most common disease affecting males worldwide, with significant variations in both incidence and mortality rates across different populations [1]. The disease has garnered significant recognition as the sixth leading cause of cancer-related mortality among males [6]. In 2018, a total of 1.2 million individuals were identified as being affected by this condition, resulting in the unfortunate death of 359,000 individuals [6]. According to a study, prostate cancer was found to be the most diagnosed cancer among males in 84 countries [7]. Furthermore, the frequency of this disease was observed to be higher in industrialized nations, while rates of development have been increasing in impoverished regions [8].

The prostate gland is an anatomical structure inside the male reproductive system that is located inferior to the bladder and surrounds the urethra. The majority of prostatic tumors exhibit a slow progression but there is a potential for cancer to metastasize to other organs, particularly the lymph nodes and skeletal system. Initially, individuals may not exhibit any symptoms whatsoever. Over time, individuals may experience symptoms such as dysuria, hematuria, or lumbopelvic pain with the progression of the condition [1]. Prostate cancer arises due to the accumulation of genetic abnormalities in prostate cells, leading to disturbances in cellular processes such as development, replication, apoptosis, and DNA repair [9]. Most prostate masses often arise within the peripheral zone, the outer part of the prostate [10]. When cellular proliferation becomes unregulated, it results in the formation of a small cluster of anomalous cells referred to as prostatic intraepithelial neoplasia (PIN).

Multiple factors, such as a familial predisposition to prostate cancer and various environmental influences, contribute to an increased susceptibility to developing prostate cancer or progressing to a more aggressive form of the disease. Inherited genetic factors contribute to around 10% of prostate cancer cases and 40% of early-stage prostate tumors [11]. Tumors may then arise from the growth of substantial PINs. Subsequently, substantial modifications often ensue as chromosomal regions undergo continuous modification or replication within the human gene pool.

To find any gaps in clinical needs and produce new ways to treat prostate cancer, it is important to have a full understanding of where it occurs, how common it is, and what it means for patients. Therefore, to enhance public consciousness regarding the potential risks associated with prostate cancer, the Obama administration officially declared September Prostate Cancer Awareness Month [12].

### Current treatment modalities and limitations

Current conventional therapies for prostate tumors include medical surgery, irradiation, and treatment with androgen deprivation [13]. Despite the demonstrated effectiveness of these medical techniques, they often come with adverse effects, limited response rates, and the eventual emergence of resistance. The resolution of these limits necessitates the exploration and advancement of alternative and complementary therapeutic approaches, a gap that can potentially be filled by the adoption of modified nanoparticle technology.

1. Medical surgery: Radial prostatectomy is a surgical procedure that involves the complete removal of the prostate gland and the surrounding tissues. The use of this treatment modality is considered a viable therapeutic option for the management of locally advancing prostate cancer [14].
2. Radiation treatment encompasses a range of procedures, which include:
  1. External Beam Radiation Therapy (EBRT) utilizes high-energy proton beams to eradicate cancer cells located in the prostate. Localized prostate cancer is frequently treated using this method, which can also be employed alongside other therapeutic interventions [15].
  2. Brachytherapy involves the localized insertion of little radioactive seeds into the prostate to deliver radiation to the tumor as a therapeutic intervention. The utilization of this approach is commonly observed in the management of localized prostate neoplasms with a low-to-moderate risk profile [16].
3. Androgen Deprivation Therapy (ADT) is a medical intervention that impedes the endogenous production of androgen hormones, including testosterone. The transduction cascade of the androgenic receptor (AR) plays a significant role in the development and advancement of prostate cancer [8].
  1. Luteinizing hormone-releasing hormone (LHRH): A luteinizing hormone-releasing hormone (LHRH) agonist is a type of medication that stimulates the release of luteinizing hormone. These drugs function by reducing the levels of testosterone, a biochemical compound that promotes the development of prostate cancer. Typically, these treatments are administered through injections or implants lasting several months or years.
  2. Anti-androgens: Anti-androgens are pharmaceutical agents that inhibit the biological activity of testosterone within the human body. They are commonly employed in combination with Luteinizing Hormone-Releasing Hormone (LHRH) agonists to augment the suppression of testosterone [17],
  3. Combined androgen and LHRH agonists blockade: The therapeutic strategy known as combined androgen blockade entails the combination of LHRH agonists with anti-androgens to achieve a more comprehensive blockade of androgen activity [18]. Enzalutamide functions as an androgen receptor (AR) antagonist, inhibiting the interaction between androgenic hormones and the AR, as well as impeding the translocation of the AR into the cell nucleus and its subsequent binding to chromosomal DNA [19]. Additional details regarding current treatment options can be found in Table 1.

**Table 1:** Current treatment modalities and their limitations

Treatment	Limitations	References
Surgery	Invasive, urinary incontinence and erectile dysfunction; limited effectiveness in advanced stages	[14]
Radiation	Though non-invasive, it possesses side effects such as fatigue, skin irritation, urinary problems, bowel problems, sexual dysfunction, and potential damage to surrounding tissues (bladder and rectum)	[15]
Androgen deprivation therapy	Resistance to ADT could arise, resulting in the advancement of the condition and adverse symptoms such as flushing, loss of sexual desire, erectile dysfunction, osteoporosis, obesity, and alterations in mood. ADT therapy spanning a considerable amount of time may raise the possibility of cardiovascular illness and problems with metabolism	[20, 21]

It is important to understand that the above constraints are not absolute for all individuals and may differ based on individual circumstances such as cancer phase, grade, and general well-being.

### Need for novel therapeutic approaches

Given the constraints of existing treatment modalities, there is an urgent need for innovative therapeutic techniques that have been shown to improve clinical outcomes, minimize adverse effects, and overcome resistance. The adoption of selenium and gold-incorporated polymeric carriers offers a promising avenue for the development of an innovative approach to prostate cancer treatment. Nanoparticle technology enables the targeted delivery of high drug loads to specific sites, hence reducing the risk of adverse effects, prolonging the half-life of the transported therapeutic component and mitigating resistance to various treatments in malignant cells [22–24]. A diverse range of nanoparticles has been synthesized and demonstrated to exhibit enhanced anticancer efficacy together with the aforementioned benefits at specific targets [25–34].

Within the realm of nanomaterials, it has been suggested that selenium nanoparticles (SeNPs) possess the highest level of promise, as they exhibit exceptional attributes for combating cancer and demonstrate favorable physiological compatibility [35, 36]. Selenium in various forms has demonstrated efficacy against cancer through different mechanisms, with a majority of them exhibiting suppressive effects on prostate cancer.

1. Selenium

Selenium (Se) was first discovered by Jöns Jacob Berzelius during the early nineteenth century. It was classified as a chalcogen and placed in group 16, alongside oxygen, sulphur, tellurium, and polonium [37]. During the 1970s, the term "selenophobia" was introduced by D. Forst to elucidate the intricate implications of selenium in the field of cancer research [38]. However, it is currently commonly recognized that Selenium (Se) has both beneficial and detrimental impacts on organisms, affecting multiple systems such as neuronal networks and the cardiovascular system [39, 40]. The integration of selenium into various chemical compounds is still a potentially safe therapeutic approach for the treatment of multiple illnesses [39, 40].

Selenium is a chemically stable nonmetallic substance that exhibits acidic properties, similar to sulphur. From a chemical perspective, Se exhibits several oxidation states, including + 2, 0, + 4, and + 6, in both inorganic and organic forms. It is worth noting that Se undergoes oxidation and reduction reactions with redox agents at a higher rate compared to sulphur (S) [41]. Hence, Se has the capacity to function as either a nucleophilic or electrophilic agent through the donation or acceptance of electrons [37]. Due to its functional characteristics, Se is deemed suitable for application in the fields of biology and chemical sciences. Selenium is found in various inorganic forms, including selenides (Se^2−^), elemental selenium (Se^0^), selenites (SeO_3_^2−^), and selenates (SeO_4_^2−^). In biological systems, selenium is present as selenocysteine and seleniomethionine [42].

Pharmaceutical chemistry, biochemistry, and biological science are interconnected disciplines that have previously recognized selenium and seleno-compounds as potential candidates for drug development. However, there has been a recent shift in attention towards selenium nanoparticles (SeNPs) as promising therapeutic options for cancer treatment. Selenium nanoparticles (SeNPs) have demonstrated the ability to induce a disruption of the cell cycle specifically during the S phase [43]. This effect is achieved by reorganizing the assembly of eIF3 proteins (Eukaryotic initiation factor 3) [44]. Notably, SeNPs exhibit a higher level of selectivity towards tumors compared to selenium Se + IV at equivalent doses [43]. Selenoproteins (SePs) are of particular interest due to their ability to modulate the functioning of various physiological systems, including the regulation of thyroid hormone synthesis, the activity of the endoplasmic reticulum, and the maintenance of the antioxidant defense system [45].

The incorporation of selenium into organoselenium compounds, whether they are synthesized or naturally occurring, often leads to beneficial pharmacological outcomes. The type of selenium bond present in an organic molecule significantly influences the likelihood of the drug exhibiting beneficial effects, such as reactive oxygen species (ROS) absorption and oxidative capacity, or detrimental effects, such as ROS induction or elemental selenium release [46].

Selenium (Se)-containing compounds have garnered significant attention in recent years due to their various possible biological effects, including but not limited to antioxidant, antibacterial, antiviral, antiparasitic, anti-inflammatory, neuroprotective, and cancer-fighting properties [47, 48].

#### Pharmacological selenium-containing compounds

**Fig. 2:**
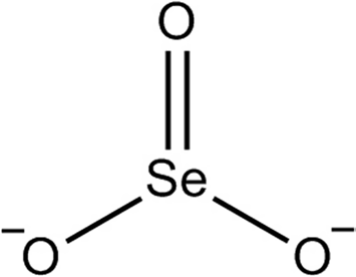
Selenite. This is adapted from [52]

It was found that inorganic compounds, especially selenite 3 (see Fig. 2 for its structure), were better at combating cancer than organic compounds [49]. This was the case even though inorganic materials exhibited increased harmful effects. On the other hand, many chemicals in the selenide, diselenide, cyanate, urea, and ester groups were not harmful and had many positive effects on cancer [50, 51]. The principal process whereby Se-containing drugs exert antitumor action in cells is cell death. Other forms of non-apoptotic cell death are an end to the cell cycle, necrosis, autophagy, ferroptosis, necroptosis, entosis, anoikis, NETosis, and mitotic catastrophe [51].

**Fig. 3:**
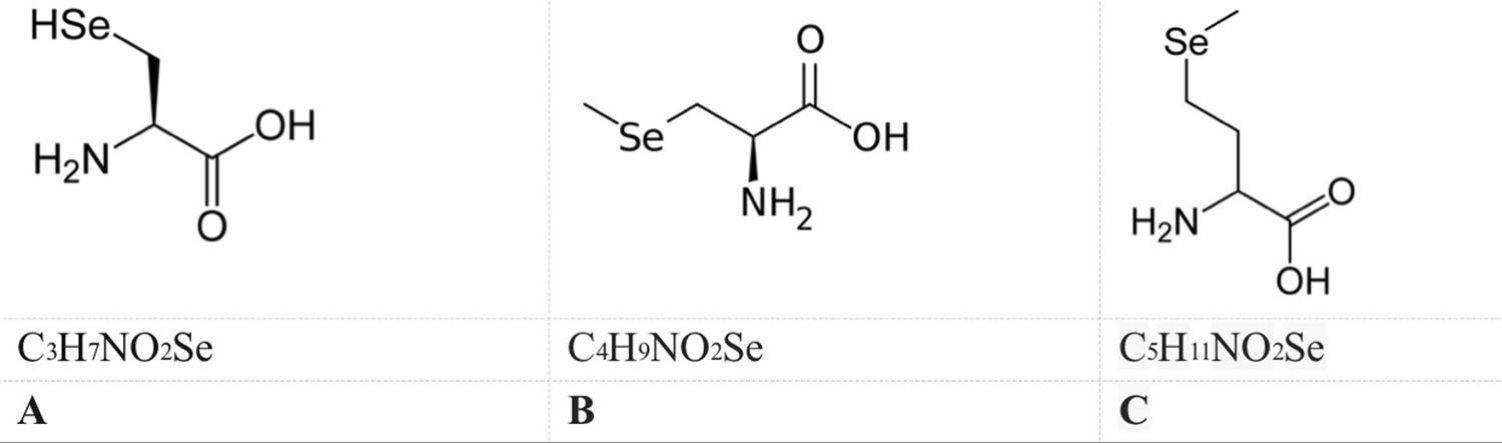
Selenocysteine (**A**), methylselenocysteine (**B**), and selenomethionine (**C**). This is adapted from [52]

Several others include amino acid-derived selenium substances (the widely investigated selenoaminoacids as chemotherapy agents are selenocysteine a, methylselenocysteine b, and selenomethionine c, (The illustration of selenocysteine, methylselenocysteine, and selenomethionine are shown in Fig. 3) which consist of a selenium atom substituted for by sulfur). According to claims, a, b, and c have activity against proliferation facilitated through an enhanced apoptotic level in certain tumor cells (IC50 greater than 100 M) [51, 53].

**Fig. 4:**
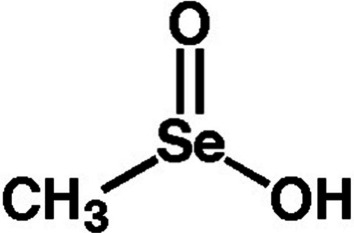
Methylseleninic acid. This is adapted from [52]

Methylselenic Acid 6 is an organo-selenium chemical formed from the oxidative breakdown of methylselenocysteine 1a [53]. Numerous cell culture investigations have shown that it has chemopreventive and antitumor characteristics in a variety of cancer cell lines, notably prostate, head & neck, blood cancers, breast, lung, ovarian, pancreatic, and esophageal carcinoma (IC50 values ranging from 1 to 40 M) [54]. Figure 4 below shows the structure of Methylseleninic Acid [52].

**Fig. 5:**
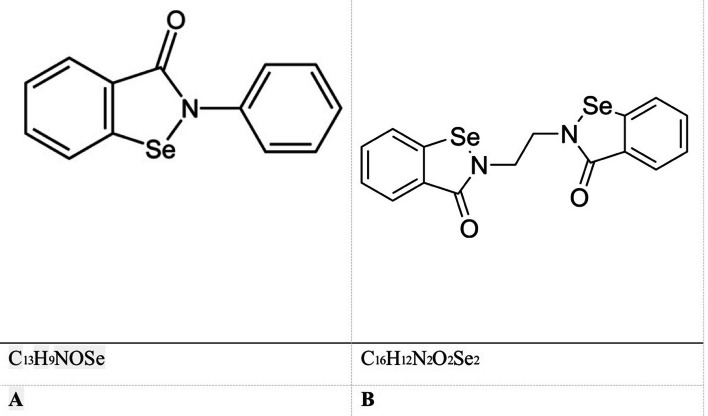
Ebselen (**A**) and Ethaselen (**B**). This is adapted from [51]

Ethaselen, a homologue similar to ebselen 4, and Ebselen, a heterocyclic molecule containing selenium as a chemical component, are the subjects of discussion. The structures are seen in Fig. 5. Numerous investigations have been conducted to investigate the medicinal properties of Ebselen. These studies have demonstrated its potential use in addressing a range of illnesses, such as bipolar disorders, hearing impairment, parasite malaria, tuberculosis, and even cancer [40, 55]. Ethaselen has demonstrated anti-cancer properties in vitro cell cultures and in vivo animal models across a diverse range of experimental cancer systems, including lung, tongue, stomach, liver, colon, prostate, cervix, nasal passages, and leukemia [47].

**Fig. 6:**
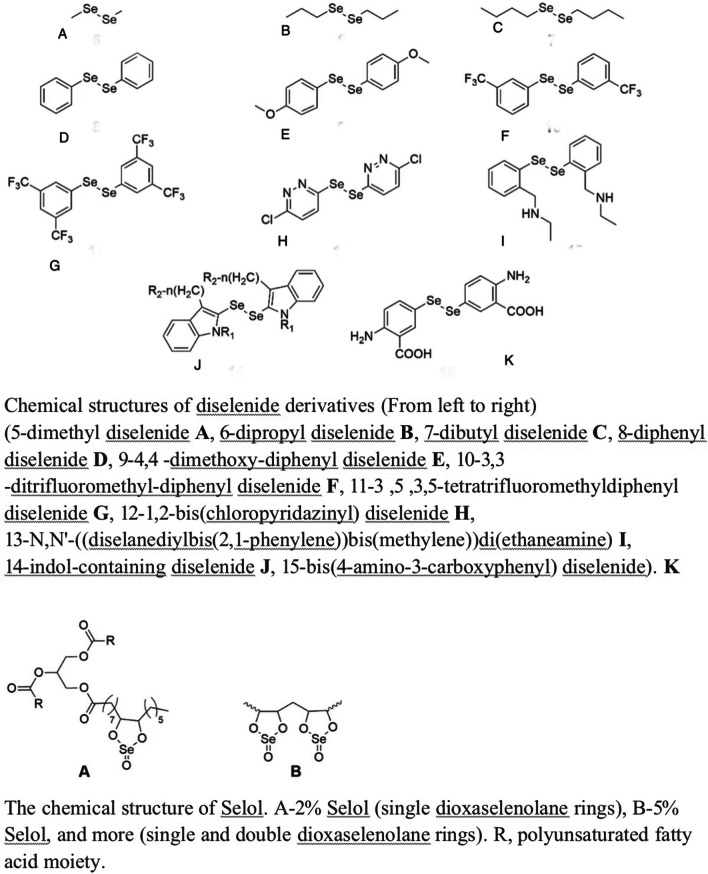
Diselenides and Selol. This is adapted from [51]

Several examples of selenium compounds include diselenides, polysaccharide-derived selenium molecules, and selol. Diselenides, selenocystamine 9 (with an IC50 value more than 10 M in the Hep cell line) and diphenyl diselenide 8 (with an IC50 value ranging from 15 to 30 M) have been linked to potential anticancer properties [47, 48]. Selenium polysaccharides are spontaneously synthesized compounds resulting from the interaction between inorganic selenium and complex sugars, hypothesized to possess a combination of characteristics derived from both constituent components. Several natural compounds were found and subjected to testing in order to determine their ability to suppress the proliferation of cancer cells in different models [47]. The formation of Setol occurred through the chemical reaction between triglycerides found in sunflower oil and selenic acid. However, the precise molecular structure of Setol remains unknown [56]. The activity of selol is solely determined by its selenium content, which is dependent upon the quantity of dioxaselenolane it possesses. Research has demonstrated that selol 2% exhibits antioxidant properties, but selol 7% has been observed to possess cytotoxic characteristics [51]. Figure 6 illustrates the chemical structures of more diselenide derivatives and selol.

Considering the similar pharmacological effects of SeNPs in combating tumors as inorganic and organic selenium, while also possessing a reduced toxicity profile, further research is being conducted on the potential of these particles as cancer therapeutics [57, 58]. The increased utilization of nanotechnology in biomedical research can be attributed to the numerous advantages it offers.

They possess inherent stability and solubility; their affinity for targeting cells can be easily engineered; they demonstrate a high biocompatibility index, a tolerable degree of immune generation, minimal adverse effects, rapid degradation, low toxicity, anticancer, and antiviral capabilities [47, 59]. This selectively protects healthy cells from cellular damage and normal cells from the cytotoxic effects of cancer chemotherapy [60, 61]. With regard to low toxicity, this has been extensively documented, in addition, it has been observed that SeNPs can be efficiently cleared from the body after cancer treatment [60, 61], hence preventing long-term adverse effects [62]. Several published literatures also support the efficacy of these substances in combating oxidative stress and their long-term toxicity [62]. Furthermore, their efficacy in combating inflammation and diseases associated with metabolism, such as cancer, diabetes, arthritis, and nephropathy, was also documented [63]. Their particles possess the capability to interact with macrophages and activate the innate immune system to decrease antimicrobial activity through the release of inflammatory cells [64].

Currently, efforts are being made to apply these properties in the field of cancer treatment. Based on the premises that have been stated so far, it can be argued that SeNPs (selenium nanoparticles) deserve the attention they are currently receiving in the field of cancer therapy.

2. Gold

Nanogold nanoparticles (AuNPs) possess biocompatible characteristics and have demonstrated efficacy in the treatment of cancer due to their enhanced penetration capabilities and sustained therapeutic effects. The different pharmacological behavior of AuNPs can be attributed to their size and shape [61, 65]. In order to alter their therapeutic properties, gold nanoparticles (AuNPs) can be fabricated in various geometries, such as hollow gold nanoshells (AuNSs), hollow gold nanospheres (AuNSs), hollow gold nanorods (AuNRs), hollow gold nanostars, gold nanoprisms (AuNPrs), gold nanoclusters (AuNCs), and gold nanoparticles (AuNP) [65]. Hence, the characteristics of the particles within the body and their optical attributes can be altered upon resizing, encompassing modifications to the charge and structure of those particles, among other factors. In addition, the functionalization of AuNPs with single or multiple ligands can enhance their ability to actively target malignant tumors [66].

Gold nanoparticles (AuNPs) exhibit excellent luminous properties due to their ability to absorb and scatter radiation, making them nanoparticles of plasmon resonance. When gold nanoparticles (AuNPs) interact with light of a specific frequency, they induce oscillations in the conductive electrons on their outer shell. This process is known as localized surface plasmon resonance (LSPR) [67]. The amounts of light absorption and scattering are determined by this phenomenon. The dimensions and morphology of AuNPs are two key factors that are correlated with the frequency of their absorption band [66]. The modulation of AuNP uptake can be achieved by the manipulation of key parameters since the optical characteristics of AuNPs are affected by their size and shape [67]. AuNPs are being researched for photothermal treatment (PTT) and ROS-producing combinations to treat cancer, according to the topic of this review.

In addition to photothermal therapy (PTT), there has been significant research conducted in recent years on the use of gold nanoparticle (AuNPs)-based nanotechnologies for combinational multimodal therapies. These therapies encompass several approaches, including photodynamic therapy, chemotherapy, radiation therapy, and immunotherapy, among others, to effectively eradicate tumor cells. It has been shown that gold nanoparticles (AuNPs) can absorb light in the near infrared region, specifically between 750 and 1400 nm in wavelength. Because they can absorb light, AuNPs can effectively turn light energy into heat, which causes a photothermal therapy (PTT) effect [68]. The Surface plasmon resonance (SPR) property described earlier is shown by gold nanoparticles (AuNPs) under near-infrared (NIR) laser which consequently leads to induction and generation of high-temperature electrons on the outer region of the nanoparticle. The energized electrons transmit the accumulated energy to the metal lattice by the emission of thermal waves, which is then dissipated through interactions between phonons. The heat produced is subsequently released into the surrounding environment [69], increasing the temperature within the cell compartment holding the nanoparticles to a range of 40–48 °C. The potential consequences of this phenomenon may lead to permanent damage to cellular structures or genetic material. The plasmonic photothermal transducers used in this study exhibit variations in their performance due to variances in the surface plasmon resonance (SPR) vibrations, which are dependent on the cross-sectional area of the structured gold nanoparticles (AuNPs) [70].

Furthermore, a diverse range of gold nanoparticles (AuNPs) exhibit plasmonic absorption when exposed to specific near-infrared (NIR) wavelengths. Within the first near-infrared radiation window, gold nanospheres show strong plasmonic absorption. Within the second near-infrared radiation window, gold nanorods also show this property. Researchers are looking for gold nanoparticles (AuNPs) with a large extinction cross-section that includes both the absorption (Cabs) and scattering (Csca) cross-sections and a high Cabs/Csca ratio. These are mostly used for photothermal therapy (PTT) [71]. Nanogold forms such as AuNRs (gold nanorods) and Au nanocages have an increased extinction cross-section and a decreased limit or threshold [72].

However, it has been discovered through research that gold nanoparticles with pointy ends exhibit superior photothermal conversion efficiency compared to other types [73]. Empirical evidence has substantiated the excellent qualities exhibited by gold nanoparticles (AuNPs), in various shapes such as nanorods, nanostars, and nanocubes [74, 75]. Additionally, compared to AuNRs, AuNPrs are more easily absorbed by cells [76]. Therefore, the utilization of AuNPrs has several benefits. One notable benefit is their enhanced safety profile, particularly when compared to smaller AuNRs, since they possess the ability to be eliminated from the system at a faster rate over an extended duration [77]. Although AuNPs of various shapes show promise as photothermal therapy (PTT) agents, their application in medicine is limited due to their weak photothermal properties. However, this limitation can be addressed by reducing the size of AuNPs and modifying their surface chemistry to specifically target tumor sites [78].

Following repeated near-infrared (NIR) exposure, it was noticed that the gold nanoparticles (AuNPs) exhibit a loss of photothermal conversion capability. In addition, it has been observed that AuNPs exhibit a limited capacity for drug loading, hence imposing limitations on their use as drug delivery vehicles [79]. It was also noticed that a considerable proportion of nanoparticles (NPs) have challenges reaching the tumor site due to the intricate and interconnected anatomical features of tumors, as well as the lack of blood vessels in the tumor region [80]. Hence, to achieve the highest level of selectivity towards the tumor site [81], selective ligands such as immunoglobulin single-chain segments of immunoglobulin carbohydrates or simpler amino chains are attached to the surface of AuNPs. Surprisingly, the active targeting of AuNPs may be influenced by changes in their shape and size, despite the presence of a targeting ligand [82].

3. AuNPs/SeNPs and prostate cancer

Gold nanoparticles (AuNPs) have been investigated as a potential vehicle for targeted delivery of anti-cancer drugs specifically to prostate tumors, with the aim of minimizing adverse effects on healthy, functioning cells. By filling AuNPs with ligands targeting prostate carcinoma cancer-specific biomarkers such as prostate-specific membrane antigen (PSMA) [81].

1. Treatment with radiation enhancement:

By functioning as radiosensitizers, AuNPs can improve the efficacy of radiation therapy. These nanoparticles can boost ionizing radiation absorption, resulting in increased DNA damage and tumor cell death [82].

2. Chemopreventive effects:

SeNPs' chemopreventive abilities in prostate cancer were studied. They can alter cancer-related signaling routes of metabolism, control the expression of genes, and have a protective effect against oxidative damage [47, 59].

3. Capability for imaging:

AuNPs also have good imaging capabilities, allowing them to be used as contrast agents in imaging contexts including computed tomography (CT) and photoacoustic imaging (PAI). This makes real-time monitoring of therapy responses possible [83].

#### Seleniuim and gold nanoparticles case studies and clinical trials in prostate cancer therapy

Despite the relative lack of emphasis on prostate cancer treatment within the expanding domain of nanomedicine, the existing research suggests a promising outlook. Selenium nanoparticles (SeNPs) have been demonstrated to exert the most significant impact on prostate cancer. In the study conducted by [84], the cytotoxicity of selenium nanoparticles (SeNPs) was examined on eight different tumors cell lines. The findings of the study revealed that selenium nanoparticles (SeNPs) exhibit a substantial inhibitory effect on patient-derived prostate cancer (CaP) cells, regardless of their androgen dependency status. According to the findings of their study [84], it has been observed that SeNP therapy facilitates the induction of apoptosis in prostate tumors through the upregulation of miR-16.

The study conducted by [85] demonstrated SeNP’s significant cytotoxic effects on PC-3 cancer cells. SeNPs were shown to be able to inhibit the proliferation of LNCaP (lymph node prostate carcinoma) cancer cells [86]. This study claims to be a very preliminary examination of SeNPs' in vitro anticancer activity to stop patient-derived CaP (prostate) cells.[84].

**Fig. 7:**
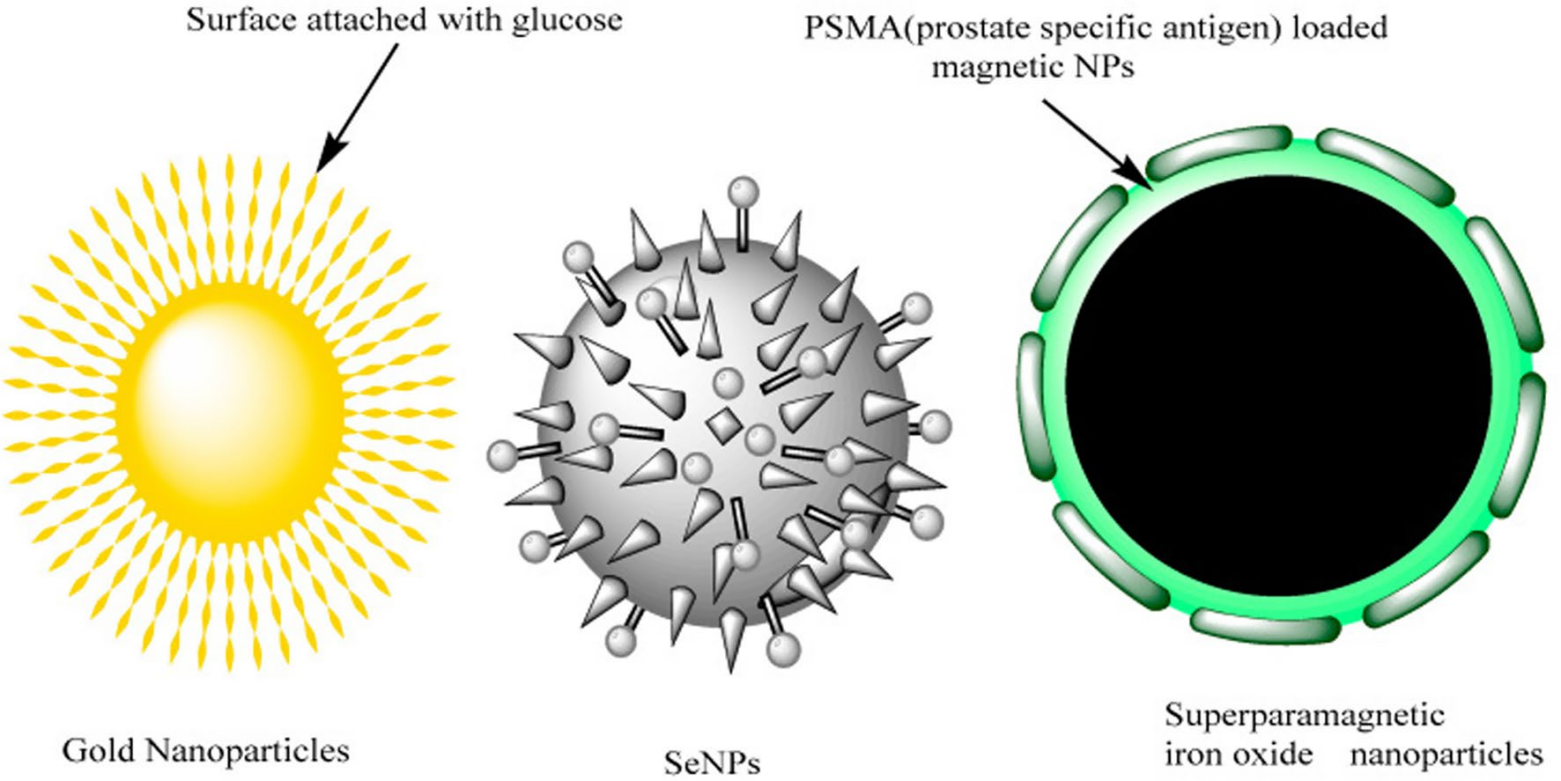
The use of glucose-functionalized gold nanoparticles, selenium nanoparticles (SeNPs), and superparamagnetic iron oxide nanoparticles was recently investigated for their potential application in the treatment of prostate cancer tumors. Adapted from [87]

Bioderived silver (Ag) and gold (Au) nanoparticles have emerged as promising therapeutic options for the treatment of prostate tumors, as stated by [87]. [87] developed glucose-coated nanoparticles with the purpose of selectively internalizing them into rapidly dividing cells. Prostate tumors cells exhibit a selective uptake of glucose-coated gold nanoparticles (referred to as Glu-GNPs) that are designed to mimic cancer cell metabolism, facilitating their transportation into the cellular cytoplasm. The schematic representation of the nanoparticles that have been modified with glucose can be found in Fig. 7 [87].

**Fig. 8:**
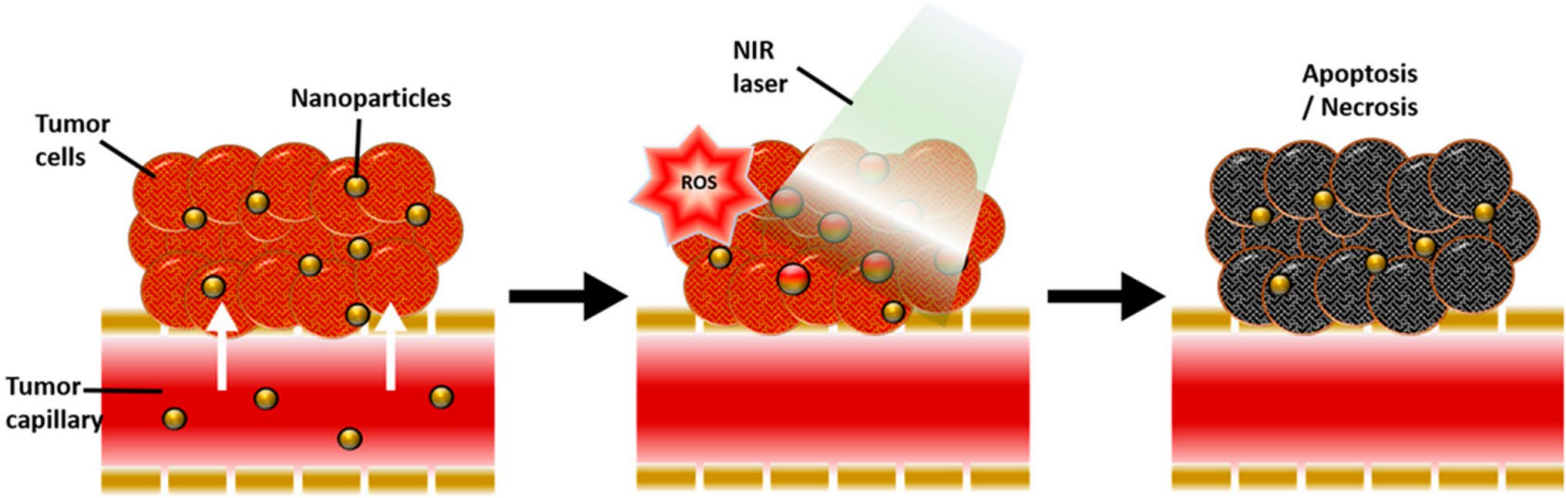
Diagram of PTT

The researchers used Au-silica nanoparticles for the purpose of conducting ultra-focal photothermal ablation as a treatment option for prostate cancer [88]. The use of Au-silica nanoparticles enables the absorption of near-infrared light at wavelengths characterized by significant matter permeability. This property facilitates a highly localized optical-focused strategy for the therapeutic intervention of prostate tumors while minimizing potential adverse effects. The anti-prostatic activity of selenium nanoparticles (SeNP) is attributed to their capacity to hinder the proliferation of rapidly dividing cells through the induction of cell cycle arrest during cellular division [89]. The use of selenium nanoparticles (SeNPs) in combating cancer induces modifications in the biomechanical properties of tumors, resulting in a reduction of the cohesive forces that maintain their structural integrity. Furthermore, the compact dimensions of SeNPs provide enhanced and specific internalization by various cell types, leading to improved drug delivery and localization at the desired spot, as depicted in Fig. 8 [63, 85].

Gold nanoparticles (AuNPs) have garnered significant interest as a promising option in the co-administration of drugs, photothermal therapy (PTT), and photodynamic therapy (PDT) in the management of prostate cancer. The ongoing clinical trials with gold nanoparticles (AuNP) highlight their potential significance in translational research and their potential efficacy in eradicating prostate tumors.

Gold AuroShell nanoparticles were assessed in clinical studies for the treatment of prostate cancer. The initial study, published in 2015, revealed the absence of any enduring adverse effects [90]. In a subsequent study conducted in 2016, a total of 16 subjects were effectively subjected to AuroShell-based laser therapy. The results indicated that after three and twelve months of treatment, 60% and 87% of the treated areas, respectively, were found to be devoid of tumors [91]. In a case report published in 2022, it was observed that the segmented tumors were completely eliminated in 100% of the cases, while the cancerous boundaries were eliminated in 94% of the cases. Currently, a multi-facility trial is being conducted to investigate the efficacy of the therapy and its applicability in the medical field. The trial involves a cohort of 45 patients who will be followed up for a duration of 12 months, aiming to provide additional insights into the therapy's effectiveness and its potential for clinical implementation [90].

The current clinical trials involving gold nanoparticles within the framework of gold and selenium photoactive nanoparticles for drug delivery by photothermal therapy (PTT) and photodynamic therapy (PDT) in the treatment of prostate cancer highlight their increasing translational significance and potential for therapeutic efficacy. (See Table 2 for a summary of current clinical trials.) These studies give us vital information about how safe, effective, and what kind of therapeutic results they have. This makes it possible for more advanced and personalized prostate cancer treatment plans to be made.

**Table 2:** Current clinical trials using gold nanoparticles in the context of gold and selenium photoactive nanoparticles for drug administration via PTT/PDT in prostate cancer treatment

Name	NP type	Application	Clinical trial	Reference
CYT6091	Tumor Necrosis Factor modified PEGylated 27 nm AuNP	Cancer treatment by immunological modulation	NCT00356980, Phase 1, Completed (2006–2009) NCT00436410, Phase 0, Completed (2006–2009)	[91]
AuroShell	PEGylated 120-nm silica core and 12 to 15-nm gold shell	Photothermal elimination of prostate tumors and other types of cancer	NCT00848042, completed (2008–2014) NCT01679470, terminated (2012–2014) NCT02680535, completed (2016–2020) NCT04240639, Recruiting (2020 –)	[90]

## Principles of photothermal therapy

### Basic concepts and mechanisms

Photothermal therapy as a cancer therapeutic technology does not invade the patient’s body as in some other treatment alternatives. It utilizes photoactive particles to convert light energy into heat, which is harnessed in malignant cell growth removal [90]. The mechanism involves the retention of light by photothermal particles; the resultant change of light energy into heat causes cell death through hyperthermia. To achieve this, near-infrared radiation, especially window I and II at 800–1250 nm is used. Due to the structural characteristics of their blood vessels, cancer cells possess a reduced ability to tolerate elevated temperatures. Hyperthermia induces cellular damage through the disruption of membranes and denaturation of proteins and nucleic acids, resulting in irreversible apoptotic effects [92, 93].

Near-infrared (NIR) light exhibits significantly lower energy levels compared to higher-energy radiation such as ultraviolet (UV) light. Consequently, NIR light possesses the ability to efficiently facilitate tissue healing and penetrate deeper into cellular structures [94]. Typically, near-infrared (NIR) light has the ability to penetrate up to a depth of 1 cm within cell tissues. It is widely recognised that the energy of near-infrared (NIR) light exhibits a positive correlation with frequency, resulting in enhanced penetration of light into the deeper layers of tissues. The depth of light penetration, typically ranging from 1 to 10 cm, is influenced by multiple factors including nanoparticle size and shape, tissue composition, near-infrared (NIR) frequency, among others [95].

Notably, only nanoparticles characterized by simple surface functionalization process, high photostability, plasmon resonance tunability, and high photothermal conversion efficiency should be used in PTT [96]. Therefore, solid LSPR AuNPs and SeNPs are recommended for use in PTT-based disease treatment [97–99].

The PTAs (AuNPs and SeNPs) absorb NIR, causing the malignant cells to become hyperthermic. Following a series of stages, this elevated temperature in the cancer microenvironment induces apoptosis. This is adapted from [94].

### Drug release mechanisms associated with microcarriers in the context of gold and selenium photoactive nanoparticles for drug delivery via PTT/PDT in prostate cancer therapy

An improved approach to drug delivery for photodynamic therapy (PDT) and photothermal therapy (PTT) in the context of prostate cancer treatment has been achieved through the incorporation of gold and selenium photoactive nanoparticles onto microcarriers. In order to enhance the precise and regulated administration of therapeutic agents, it is critical to understand the various drug release mechanisms associated with these microcarriers.

1. Temperature-controlled release

Adding gold nanoparticles to the microcarrier matrix makes it possible for drugs to be released by photothermal means (carrier shrinkage) when the temperature rises. When gold nanoparticles are exposed to near-infrared light, they cause localized hyperthermia. This can change how therapeutic payloads are released, leading to a precisely controlled and on-demand drug delivery profile [100, 101].

2. Redox-responsive release

Using selenium-based nanoparticles in microcarriers to increase the levels of reactive oxygen species (ROS) inside cells can help release drugs that is responsive to redox conditions. Due to their redox-sensitive properties, selenium nanoparticles can release therapeutic compounds precisely when prostate cancer cells are under oxidative stress [102]. This redox-responsive drug delivery method uses intrinsic oxidative stress pathways to make PDT more effective and provide a personalized and targeted drug delivery strategy [103].

3. Enzyme-triggered release

Enzyme-responsive scaffolds in microcarriers can produce enzyme-triggered drug delivery in the tumor’s milieu. Overexpression of enzymes in the tumors surroundings can accelerate the destruction of the delivery system matrix, resulting in the regulated dispersion of drug contents [104]. This enzyme-activated delivery mechanism enables targeted drug distribution to the tumors site, reducing off-target effects and increasing the precision of PTT/PDT in prostate cancer therapy [105].

4. Light-activated release

Photoactive materials packed into the microcarrier design allow for light-triggered drug release in response to certain wavelengths of light utilized in PTT and PDT. When exposed to light, photo-sensitive binders or cross-linkers can be cleaved or undergo re-structuring, promoting the release of packed drugs [106]. This light-triggered delivery mechanism allows for fine spatiotemporal regulation of drug administration, enabling personalized and targeted treatments for prostate cancer [101].

5. pH-dependent release

Microcarriers made of pH-responsive materials can be used to release drugs in an acidic environment inside a tumor. The acidic pH of cancer cells could change their structure or destroy pH-responsive delivery platforms, which would allow cancer drugs to be released in a controlled and targeted way [107]. This pH-sensitive discharge mechanism allows for tailored drug distribution in response to the physiological circumstances of prostate cancer tumors, hence increasing the efficacy of PTT/PDT [108].

6. Diffusion-controlled release

This is a basic mechanism found in numerous microcarrier systems. The variation in concentration and the diffusion coefficient of the drug inside the carrier matrix primarily influence the drug release rate [109]. The porosity and size of the drug molecules, as well as the qualities of the carrier material, all have a substantial impact on the diffusion process [110].

7. Degradation-controlled release.

Certain microcarriers contain biodegradable polymers that decay over time, allowing enclosed drugs to be released gradually. The degradation-controlled release approach enables fine control of release kinetics and drug delivery duration [111]. The breakdown rate of the polymer and the stability of the medication within the carrier matrix are two factors that influence this process [112].

8. Stimulus-responsive release

For drugs to be released from microcarriers in a stimulus-responsive way, they have to be released when certain environmental stimuli are present. Changes in pH, temperature, or enzyme activity can be used to control therapeutic drug release in the target region [113]. This technique has the benefit of allowing for targeted and on-demand medication release, reducing off-target effects, and increasing therapeutic effectiveness [114].

The nanoparticle delivery pathway for photoactive selenium and gold nanoparticles must be understood in order to develop systems for delivering pharmaceuticals to individuals with prostate cancer in a targeted and efficient manner. Enhancing the delivery method of nanoparticles in order to optimize the targeting and efficacy of PTT/PDT paves the way for the development of more effective and individualized treatment approaches.

### Clinical applications of AuNP-based PTT for prostate cancer therapy

The distinct optical features and strong biocompatibility of gold nanoparticles (AuNPs) make them potential therapeutic platforms for targeted and image-guided treatment in cancer therapy.

1. Role of gold and selenium photoactive PTT/PDT nanoparticles in drug delivery in prostate cancer therapy:

Gold and selenium photoactive nanoparticles have become flexible ways to deliver drugs when used with both photodynamic therapy (PDT) and phototransduction therapy (PTT). These nanoparticles absorb alight in the near-infrared range, allowing for an effective conversion of light energy into heat and producing localized hyperthermia at the tumor’s location [115]. Also, selenium nanoparticles have redox properties that let reactive oxygen species (ROS) form. This makes photodynamic therapy a conceivable way to treat cancer [116].

2. Enhanced targeting and selective treatment efficacy:

These tiny gold particles and selenium photoactive particles make it easier to target and treat prostate cancer when they are used as drug delivery platforms in PTT and PDT. These nanoparticles may be functionalized by targeting ligands such as antibodies or peptides to allow for targeted interactions with cancer cells, reducing off-target effects and increasing therapy results [117]. The synergistic effects of PTT and PDT improve the treatment regimen's therapeutic effectiveness even more [108].

3. Clinical trials and translational potential:

Several preclinical studies have shown that AuNP-based PTT is a safe and effective way to treat prostate cancer. Using AuNPs along with PTT/PDT in clinical studies is currently being carried out to study treatment response, disease progression, and long-term outcomes in men with prostate cancer [90]. These techniques' translational potential offers promise for the development of tailored and targeted systems in clinical practice.

### Advantages and challenges in cancer treatment

Photothermal treatment offers a few benefits, including precise tumors targeting, negligible obtrusiveness, and the potential for combinatorial therapy. In any case, issues involving restriction of penetration of light by tissues, loss of heat, and harm to surrounding cells and tissues should be monitored. New AI and computational approaches may offer even more optimization [113]. This is summarized in Table 3 below.

**Table 3:** Summary of advantages and challenges of PTT in cancer treatment

Advantages	Challenges
Photo-responsive materials are localized in the tumor area. [118, 119] Targeting ligands such as antibodies, single-chain fragments of antibodies, carbohydrates, etc. can be furnished on AuNPs to combat low targeting ability [81]	Low targeting ability due to the hindrance caused by the dense interstitial structure of the tumor and lack of vessels in the tumor [80] and defects of photosensitive materials [120]
Produces high local temperatures with no or minimal influence on healthy cells and tissues [119]	Possible thermal damage to normal tissue [120]
PTT can be combined with other types of therapies with different mechanisms of action, which results in a synergetic treatment effect [5, 88, 121]	Insufficient photothermal effect and limited penetration depth of light in biological tissues [88, 120]
Upgradable thermal capacity is achieved by manipulating the size of gold nanoparticles and modifying their surface properties for the targeted location of the tumor [78]	Poor photothermal stability [78]. Can lose their photothermal conversion ability upon repetitive NIR radiation [79]
Compensate for poor drug loading capacity with remarkable light-absorbing and scattering abilities [67]	AuNPs have a poor drug loading capacity, limiting the use of AuNPs as drug carriers [79]

The main benefit of PTT is that the photo-responsive materials only reach the tumors, raising the temperature without having much of an effect on the healthy cells and tissues nearby [118]. The selective spatiotemporal nature of PTT, the way it affects immunogenicity, and the fact that cancer cells cannot avoid it shows its usefulness [122]. Furthermore, PTT can be synergized with a variety of other therapeutic approaches, which may include surgery, chemotherapy, immunotherapy, etc. [123].

PTT exhibits some drawbacks that must be addressed to ensure smooth clinical integration. These are: (a) poor focusing capability and photoactive agent flaws; (b) weak photothermal impact; and (c) potential heat harm to healthy cells [120]. PTT has the potential to be applied to a wide range of malignancies that can be targeted with an electromagnetic pulse (laser radiation) within the tumors. This includes soft tissue tumors [89], prostate malignancies [124, 125], and tumors in the cervical region [126]. The efficacy of unfurnished photoactive compounds in targeting tumours is contingent upon their ability to permeate while maintaining the EPR effect [89].

Nanoparticles (NPs) with a 100-nm diameter are capable of penetrating cancerous cells and persisting for an extended period of time [127]. Although the entire amount of gathered NPs for administration via IV does not surpass 5–10%, which is insufficient to create an elevated level of nanoparticles inside of a tumor adequate for PTT [127], Intratumoral injections are frequently used to boost the level of photothermal materials inside the tumor. In fact, the harm caused by PTT-induced heat overload and minimally invasive near-infrared (NIR) radiation to healthy tissues can be decreased by localized injection of photothermal materials in the tumor’s location [121]. Micrometric objects are typically used to reduce the dispersion of therapeutics off targeted cancerous sites, especially within the large vital organs (lungs, liver, etc.). Focused therapies for cancer are currently delivered via small spheres or tiny caps utilized for healthcare and biomedical research. This approach to drug delivery is significantly safer for patients compared to intravenous injection, while also delivering a greater quantity of biological agents to the tumor [120, 121].

Another limitation of PTT is that it has a restricted level of light absorption in tissue structures. As a result, PTT alone fails to be effective enough to eliminate a tumor, resulting in its regrowth and the development of malignancies throughout the human organism [88]. In order to mitigate this issue, PTT may be combined with a variety of other therapy options that operate through distinct mechanisms, thereby producing a collaborative therapeutic effect. For instance, photoactive particulates can be encapsulated with chemotherapy drugs to enable integrated PTT and medication therapy [88, 121].

### Recent advances in photothermal agents

Gold nanoparticles (AuNPs) have gained recognition as valuable photo-active agents due to their distinctive characteristics, including their ability to interact with light, surface plasmon resonance, and ease of modification qualities [65]. The incorporation of gold nanoparticles (AuNPs) into polymer-based nanocarriers enhances the structural integrity of the core, enables precise control over dosage, and facilitates accumulation within tumor tissues [61]. Furthermore, SeNPs have demonstrated capability as ROS generators and photothermal ignitors [5].

Considering the fact that the utilization of gold nanoparticles (AuNPs) and selenium nanoparticles (SeNPs) in photothermal therapy (PTT) has the potential to elicit an immune response against tumors within the human body, it is reasonable to strategically combine this approach with antibody therapy. This can be achieved by administering immune-stimulating drugs, such as PD-1 immunological checkpoint inhibitors, in conjunction with the aforementioned nanoparticles [5].

An alternative approach to enhancing the efficacy of a Photothermal therapy (PTT) agent involves combining two or more PTT agents. This development is expected to enhance the photothermal efficiency of photoactive materials. The aforementioned materials include carbon-based materials such as graphene and carbon dots, as well as semiconductor nanoparticles like CuS, CuSe, and MoS_2_. Additionally, rare earth elements such as AuNrs, Au nanoplates, and Au–Ag alloys are included in this category [118], and certain substances classified as natural agents [5]. The integration of several components into a single delivery system is anticipated to enhance the current limited photothermal reliability, as opposed to the use of a singular photothermal reactive material. Moreover, in cases where a monotherapy approach using a single-agent photothermal therapy (PTT) fails to achieve total tumor eradication, the incorporation of a second effective therapeutic agent within the suggested carriers could potentially enhance the tumor-ablation capabilities, leading to the development of a single multimodal administration system [5].

A substantial number of studies on microcarriers made up of various inorganic and organic nanoparticles have been conducted in recent years [118]. The most effective delivery systems for therapeutic purposes consist of multilayered polymer shells; biologically active chemical packaging is one example. [103]. By using a layer-by-layer methodology, numerous nanostructured PTA, can be incorporated into a single medium. In addition, small capsules containing iron oxide nanoparticles, carbon nanotubes, and silicon dioxide nanoparticles have been effectively utilised as PTAs [127]. The key points of this discussion are succinctly summarized in Table 4.

**Table 4:** Summary of recent advances in photothermal agents

Recent advances	References
Incorporating gold nanoparticles into polymer-based microcarriers enables improved stability, controlled release, and enhanced tumor accumulation	[65]
Selenium nanoparticles have shown potential as ROS generators and sensitizers for photothermal therapy	[113, 123–125]
AuNPs and SeNPs in PTT can induce the immune system, where antibody therapy can be paired with the body's natural anticancer defenses by also administering immune boosters, such as the immune checkpoint inhibitor PD-1	[5]
Integrating more than one photothermal agent will boost the photosensitive molecules' photothermal conversion. (For instance, gold nanorods, nanoplates, and gold-silver alloy), semiconductor NPs (for instance, CuS, CuSe, and MoS2), and carbon nanomaterials (for instance, graphene, carbon dots)	[5, 118]
AuNPs can enhance the permeability of reactive oxygen species (ROS) in tumor tissues, facilitated by SeNPs, which, in high dosages, could eradicate tumor cells	[5, 113, 123–125, 125]
Computational and use of machine learning in replication and simulation of the thermal reaction of AuNRs with SeNPs, microcarrier and delivery design, and overall process monitoring	[5]

Gold nanoparticles (AuNPs) of various shapes are among the most widely recognised photoactive nanoparticles for PTT, according to previous studies. This is due to their high surface plasmon resonance and ability to convert light into heat [128]. Interestingly, the thermal energy generated by AuNPs has the capacity to enhance the permeation of reactive oxygen species (ROS) into tumor tissues, thereby potentially facilitating the efficient destruction of tumor cells [88]. Consequently, utilizing ROS-responsive materials and increasing ROS levels in cancer cells promoted apoptosis [88]. One potentially effective strategy for inhibiting tumor growth involves the synergistic interaction of materials mediated by ROS and PTT. However, multiple sources assert that the fundamental reason for the increased heat production by a mixture of AuNRs and SeNPs has not been fully identified and explained [113].

According to a study [5], A computational simulation of the thermal reaction between AuNRs and SeNPs was first conducted, then the researchers evaluated the polymer capsules containing the mixed SeNPs and AuNRs for B16-F10 mouse cutaneous cancer tumors treatment. This system exhibited enhanced combined ROS-mediated photothermal properties when subjected to a focused laser beam, both gold nanorods (AuNRs) and selenium nanoparticles (SeNPs) had an increase in thermal emission. Selenium nanoparticles (SeNPs) have the ability to induce tumor cell death through the generation of reactive oxygen species (ROS) at sufficiently elevated concentrations [5, 118, 119].

Currently, the design of drug delivery systems, in the field of material science and development, mainly depends on the researchers' intuitive judgement and traditional trial and error methods. AI has the potential to automate and make processes like nanoparticle design and optimization more efficient. This includes tasks such as modelling, stability assessment, and efficacy analysis. In addition, AI can enhance real-time cell imaging, provide predictive analysis for drug release kinetics and toxicity, monitor biomarkers and drug levels in real-time to enhance personalized therapy and facilitate multiscale modelling for drug delivery systems [118, 119].

## Principles of photodynamic therapy, roles of reactive oxygen species (ROS) in cancer treatment

### The biological significance of ROS

Regarding cancer, oxygen compounds known as reactive oxygen species (ROS) operate as both pro-survival messengers and lethal actors [128]. ROS are engaged in cell signaling mechanisms that participate in the body’s normal processes at appropriate levels. On the other hand, excess ROS generation can lead to oxidative stress, which can damage DNA, lipids, and proteins through oxidative breakdown, and kill cells.

Biological SeNPs derived from Bacillus *lichen* induced non-apoptotic cellular mortality in the Cap culture (PC-3) at 2 g selenium per mL, according to [85]. This was achieved by initiating necroptosis through reactive oxygen species (ROS) and obtaining cellular internalization in the cancer lines. Actual-time qPCR profiling revealed an upward trend in the circulating levels of necroptosis-dependent tumor necrosis factor (TNF) and interferon regulatory factor 1 (IRF1). Translational synthesis of the RIP1 protein (Receptor-interacting serine/threonine-protein kinase 1) was increased in response to SeNP administration. Additionally, cell survival was enhanced by the presence of the necroptosis inhibitor Necrostatin-1 [85].

### Photodynamic therapy and the potential photosensitizers utilized in PTT/PDT therapies

Photodynamic therapy (PDT) has emerged as a promising non-invasive therapeutic modality for the treatment of prostate cancer. In photodynamic therapy (PDT), certain wavelengths of light activate photosensitizers, which produce singlet reactive oxygen species (ROS). These ROS have cytotoxic effects on cells and destroy tumors. To induce ROS in tumors, many techniques have been devised, including PDT, sonodynamic therapy, and CT [103]. Given that cancer cells are more susceptible to oxidative stress than healthy cells, ROS production can be used to specifically attack cancer cells. Utilizing reactive oxygen species (ROS) in therapy presents an unprecedented opportunity to effectively treat cancer. Understanding the diverse array of photosensitizers utilized in PDT is crucial for optimizing treatment efficacy and minimizing adverse effects [108].

1. Basic concepts and mechanisms of photodynamic therapy

Photodynamic treatment operates on the principle that triplet states result when photosensitizers are activated by light. By gaining energy, molecular oxygen generates extremely unstable singlet oxygen species and other reactive oxygen species. These induce oxidative damage to cancer cells [124, 125]. This process initiates immune-mediated reactions, vascular disruptions, and apoptotic pathways, all of which aid in the shrinkage of the tumors [129].

2. Types of photosensitizers for photodynamic therapy

In order to promote personalized therapy approaches and enhance clinical efficacy in prostate cancer PDT, it is critical to comprehend the various properties and mechanisms of action of these photosensitizers.Several photosensitive substances have been studied for their effectiveness in prostate cancer PDT. These photosensitizers have different absorption and emission spectra, which allow for selective activation at certain light wavelengths. Photosensitizers that are extensively used include:

1. Porphyrin-based Photosensitizers: Due to their good light absorption qualities and physiological compatibility, porphyrin derivatives such as protoporphyrin IX (PpIX) and its precursors, Aminolevulanic acid (5-ALA) and Motexafin Lutetium (Mlu) demonstrated encouraging results in PDT for prostate cancer, with efficacy in murine, canine tumor models and human trials. These photosensitizers are effective tumor-targeting agents that may be delivered systemically or topically [130].
2. Photosensitizers based on Chlorophyll: Chlorophyll derivatives have received interest for their prospective uses in prostate cancer PDT. These photosensitizers are promising candidates for cancer treatment because they have good photochemical stability and produce phototoxic effects when exposed to light. Examples include Pheophorbide [131].
3. Photosensitizers based on Phthalocyanine: Phthalocyanine derivatives such as silicon phthalocyanine (Pc4) absorb light very strongly in the near-infrared range, which makes it easier to reach deeper into tissue and target tumors. They are interesting candidates for PDT in prostate cancer treatment due to their excellent photostability and efficient ROS production [132].
4. Photosensitizers under development: Researchers are working on new photosensitizers that are different from the ones that are already available. These include nanoparticles, carbon-based compounds, and organic dyes. Examples include TOOKAD; a vascular-targeted padeliporfin, porfimer sodium, verteporfirin, talaporfin and redaporfin (bacteriochlorin). They are being studied for use in prostate cancer PDT. The targeted specificity, biocompatibility, and photophysical properties of these new photosensitizers can all be changed [133].

### Synergistic effects of ROS, photothermal therapy, and drug release mechanisms associated with microcarriers

Coupling ROS production with PTT has been shown to have collaborative benefits for cancer cell elimination. The photothermal effect causes thermal shock, which increases the generation of cellular ROS. ROS consequently increase hyperthermia's damaging impact by worsening cellular stress and activating cellular death cascades [132]. ROS-mediated therapy combined with photothermal therapy has the potential to improve the effectiveness of treatment.

Selenium exhibits antioxidant properties at moderate concentrations; however, when present in excess, it transforms into a pro-oxidant, thereby enhancing the production of reactive oxygen species (ROS). Cancer cells exhibit susceptibility to additional reactive oxygen species (ROS) despite the high abundance of these species and reducing agents within them. [132, 134].

**Fig. 9:**
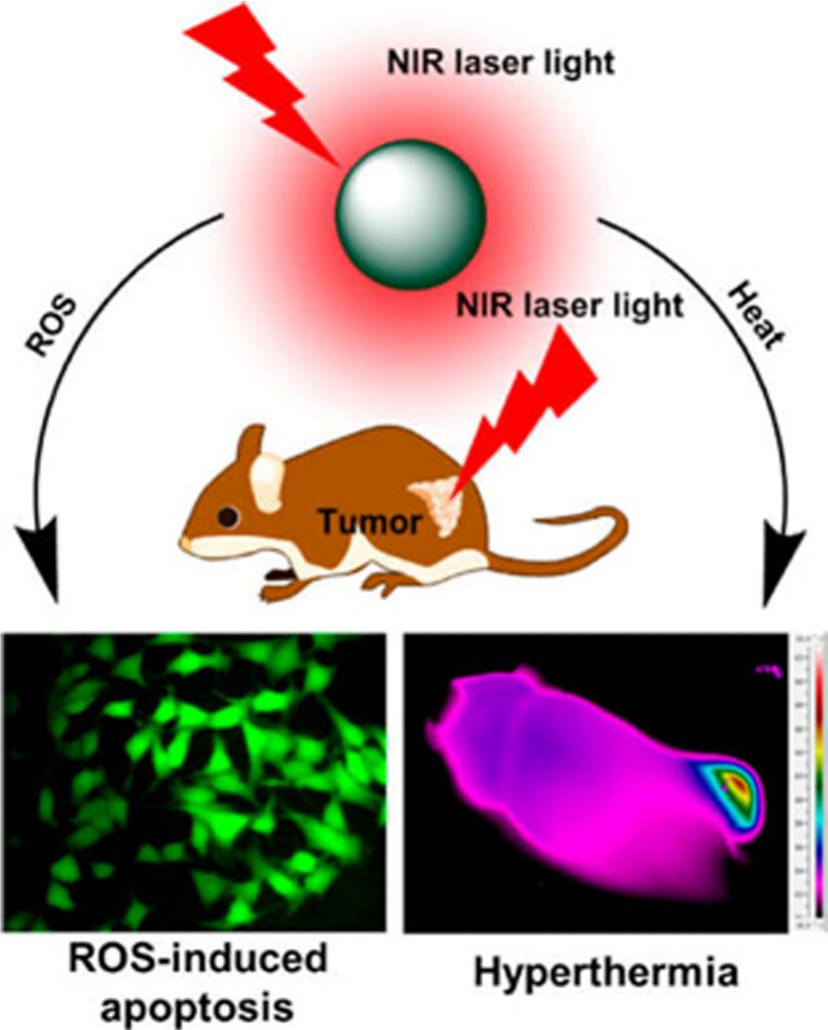
Diagram of PTT/ROS collaborative benefits for cancer cell elimination. Adapted from [134]

It was demonstrated in [5] that combining SeNPs and AuNRs within a polymer capsule enabled the development of multimodal delivery systems with a more effective combined PTT against the proliferation of melanoma tumors. To produce hybrid polymer capsules, SeNPs and AuNRs were integrated into multilayered polymer capsules. SeNPs function as reactive oxygen species (ROS) generators, while AuNRs serve as nano-heat sources. Figure 9 shows a diagram of the synergistic effects of ROS and photothermal therapy.

### Nanoparticle delivery pathway in the context of gold and selenium photoactive nanoparticles for drug delivery via PTT/PDT in prostate cancer therapy

Knowledge of the nanoparticle delivery pathway in the context of gold and selenium photoactive nanoparticles is critical in developing targeted and efficient drug delivery systems for prostate cancer therapy.

1. Better Permeation and Retention Effect: The ability of gold and selenium nanoparticles to adhere to tumor cells is due to their favorable physical and chemical characteristics, which confer permeability and retention (EPR) effects. The passive accumulation of nanoparticles in the tumor microenvironment can occur due to the organization of tumor vessels and the compromised functionality of lymphatic drainage [135]. Drugs must be concentrated exclusively in tumors in order to reduce their systemic toxicity and increase their therapeutic efficacy; thus, the EPR effect is crucial [136].
2. Receptor-Mediated Endocytosis: Nanoparticles composed of selenium and gold have the potential to enhance the specificity of ligands or antibodies that target prostate cancer cells with an excessive number of receptors. This receptor-mediated endocytosis enables nanoparticles to enter cancer cells, enabling precise delivery of therapeutic cargo [137]. Using receptor-mediated pathways can make drug delivery more specific and effective, which can lead to better therapeutic effects [138].
3. Intracellular Transport and Subcellular Localization: Gold and selenium nanoparticles are transported within cells via intracellular trafficking pathways. As a consequence, they accumulate within subcellular constituents such as endosomes and lysosomes [139]. PTT and PDT must be capable of penetrating cells effectively in order to function properly in prostate cancer cells [140].
4. Reactive Oxygen Species-Mediated Cytotoxicity: Nanoparticles made of selenium can make cancer cells produce reactive oxygen species (ROS), which can lead to oxidative stress-induced cytotoxicity and cell death through apoptosis. This ROS-mediated cytotoxicity is critical to the therapeutic effectiveness of selenium nanoparticles when combined with PTT/PDT, emphasizing the importance of the nanoparticle delivery mechanism [141].
5. Heat-Mediated Tumor Ablation and Apoptosis: Gold nanoparticles exhibit remarkable light-to-heat conversion properties when subjected to near-infrared light, thereby facilitating accurate and focused hyperthermia within prostate cancer tumors. The selective destruction of cancer cells while preserving healthy tissues in close proximity can be achieved by heat-mediated tumor ablation and induction of the apoptotic pathway via PTT [142].

## Evaluation of therapeutic outcomes: tumor regression, cell death, and treatment response of ROS-mediated (PDT) combined photothermal therapy for prostate cancer

Several metrics are employed to evaluate the therapeutic efficacy of combined photothermal therapy with ROS delivered via selenium and gold-based systems. Tissue shrinkage is assessed through the methodical monitoring of temporal variations in both tumor mass and size. Flow cytometry and immunohistochemistry can be employed to investigate cell death pathways encompassing necrosis and apoptosis. To determine the efficacy of a treatment, alterations in biomarkers that indicate the cancer's stage of progression and drug resistance are monitored [143].

The mechanisms by which ROS-mediated combination photothermal therapy is a more effective treatment have been the subject of increased exploration. The negative consequences consist of the degradation of nucleic material, oxidative stress induced by reactive oxygen species, and disruption of cellular processes that are essential for the development and survival of the tumor. The discovery of the link between ROS production and PTT [143, 144] has further defined the harmful effects of elevated temperatures and ROS-induced cytotoxicity on prostate cancer cells. Evaluation of the treatment outcomes of Au/SeNPs in PTT/PDT (ROS)/PC therapy and additional case studies are presented below.

**Fig. 10:**
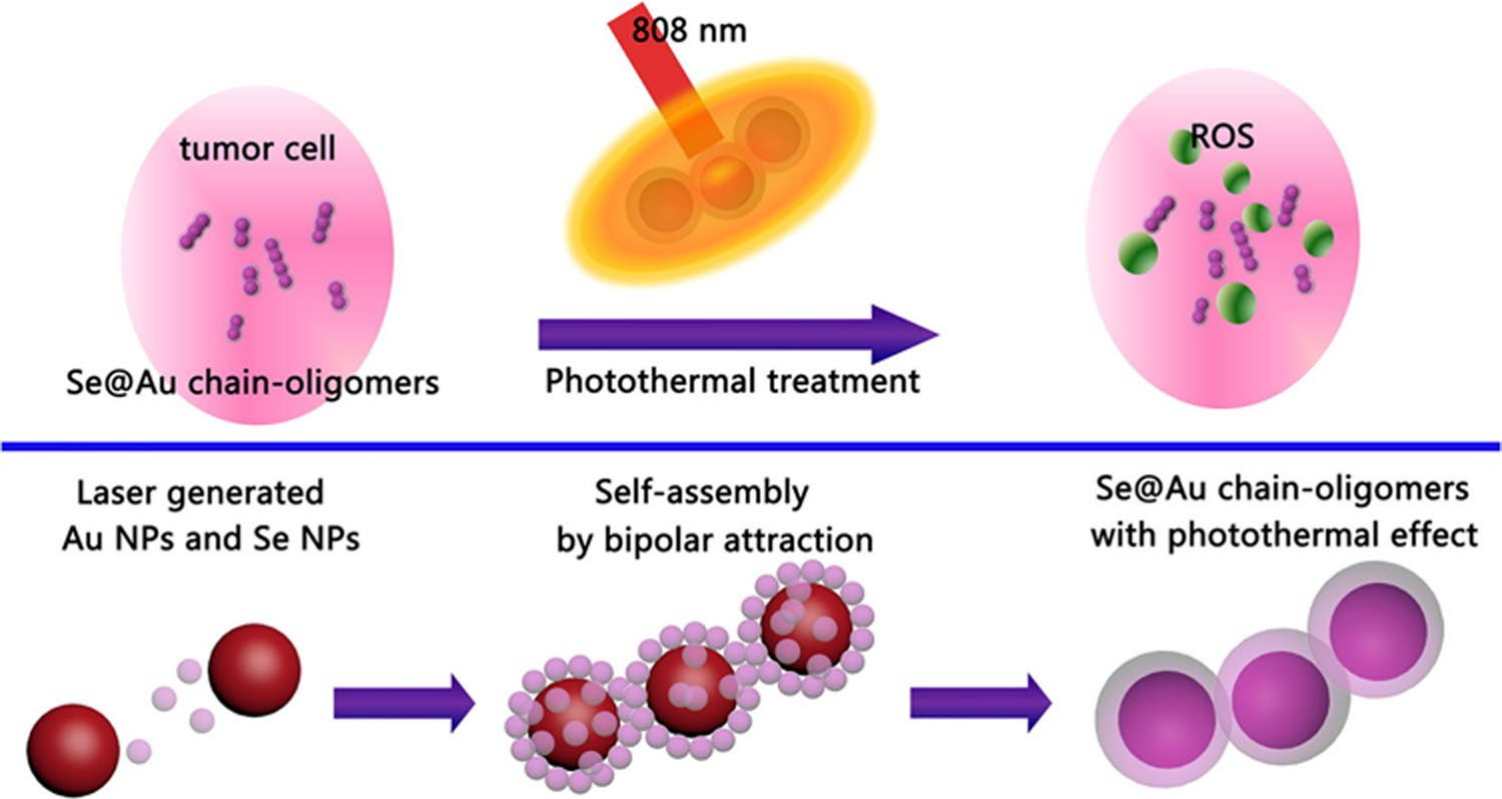
Blended non-crystalline selenium packaged in a gold chain-oligomers. This is adapted from [143]

Blended non-crystalline selenium encapsulated in gold chain oligomers was described in [143]. The Se-Au chain oligomers' synergistic microcarrier demonstrated significantly enhanced near-infrared (NIR) absorption and an initial-rate photothermal conversion efficiency (η), which subsequently improved by 47.5% at 808 nm. The analysis of their cell culture confirmed its high-quality potential. Gold-selenium was repeatedly laser-blasted in NaCl without the formation of any toxic precursor molecules. Further breakdown of this concept is presented in Fig. 10 [143].

The Se-Au chain oligomers were discovered to increase the concentration of reactive oxygen species (ROS) by 36% whenever the readings were compared before and after the NIR irradiation. The photothermal properties of the gold-selenium chain and Se's ability to combat cancer could work together to increase the concentration of reactive oxygen species between cells. Consequently, these exceptionally stable Se@Au chain-oligomers have a promising future as in vivo photothermal therapy agents for prostate cancer and other forms of the disease [143].

In a detailed study [140], several types of photothermal and photodynamic treatments were looked at as possible innovative technologies that could damage Cap's growth cells and eventually kill them. A different study [145] examined the potential of hexadecyl trimethyl ammonium bromide (CTAB) deposited on hydrophobic gold nanorods (AuNRs) to form a synergistic PTT/PDT with polycaprolactone (PCL) and indocyanine green (ICG). Photosensitizer-capable ICG incorporated into polymeric vehicles can generate reactive oxygen species and undergo photothermal modification when subjected to laser blasting. The efficacy of the PDT induced by the generated reactive oxygen species (ROS) completely destroyed the integrity of the lysosomal layer of prostate cancer cells and accelerated the demise of PC3 cells. The prostate-specific membrane antigen (PSMA)-negative emasculation-safe subtype exhibited this characteristic in particular [145]. This interaction leads to the generation of reactive oxygen species, which in turn regulates the at which chemotherapeutic agents are released. Consequently, this process results in the depletion of cell integrity, induction of apoptosis, eradication of tumours, and removal of androgen-resistant prostate cancer [145].

Beyond prostate cancer, [1] described their work on human lung cancer culture and NIH-3T3 (a mouse fibroblast cell line). Upon directing NIR laser pulses towards the experimental setup, selenium acid and lauric acid, which were previously stacked separately into Au nanocages, reacted, producing selenite. The selenite particles attack the mitochondria in human cancer cell lines killing them. This offers a promising prospect that holds great promise for synergistic cancer treatment, including prostate cancer [1].

## Design and synthesis of selenium and gold-incorporated microcarriers and deliverysystems

### Design and synthesis of microcarriers

The development of microcarriers composed of gold and selenium-containing polymers may improve the delivery of medications to prostate cancer tumors. These biologically compatible polymer frameworks can be designed to encapsulate gold and selenium nanoparticles. Particulate size, surface furnishing, and controlled release capabilities are deliberate design considerations to optimize the accumulation of drugs in tumors and guarantee treatment effectiveness [1, 5, 143–154]. Remarkable redox characteristics of selenium nanoparticles allow for the creation of ROS and the control of redox signaling pathways. They may serve as photothermal therapy sensitizers, enhancing the damaging impact of hyperthermia [147]. The significant NIR absorption of gold nanoparticles, on the other hand, makes photothermal reactions possible. They can accurately regulate the production of heat and localized hyperthermia due to their surface plasmon resonance (SPR) [147].

After identifying the techniques and resources that other researchers in related subjects have found to be most effective, the selection of suitable materials for the microcarriers and delivery systems is made using simulation and machine learning in combination with literature. Mthematical simulations, machine learning, and literature are all used together. For example, biodegradable polymers, silica, or materials based on carbon can be used as microcarriers, and gold and selenium nanoparticles can be added because of their special qualities [148]. A suitable technique is then used to generate selenium and gold nanoparticles with precise control over size and form. Several methods can be used. They may include chemical reduction, sol–gel, or green synthesis employing plant extracts [149]. The synthesized gold and selenium nanoparticles are then incorporated into the microcarriers. This could entail surface functionalization of the microcarriers [5, 87].

1. Material Selection: Depending on the intended application, the choice of material for microcarrier synthesis is essential. To ensure low toxicity and immunological reactions, biodegradable and biocompatible materials are frequently used for biomedical applications [150]. Common materials include biodegradable polymers like poly (lactic acid), poly (caprolactone), and poly (lactic-co-glycolic acid). Compounds like silica, carbon-based compounds, or metal oxides may be employed for additional applications [150, 151].
2. Solvent Selection: Choosing the right solvent is crucial for the synthesis process since it affects the morphology and size of the microcarrier and dictates how easily the components will disperse [152]. Organic solvents like dichloromethane, chloroform, and dimethylformamide (DMF) are examples of common solvents. Solvents that are water-miscible, such as ethanol or acetone, can be employed with water-based systems [152].
3. Techniques for Synthesis: The method chosen for microcarrier synthesis relies on the material, the desired size, and the morphology [151]. Typical strategies include:
  1. Emulsion/Solvent Evaporation: In this process, the polymer is mixed with a stabilizer in an aqueous phase. This is done after the polymer has been dissolved in an organic solvent. The subsequent evaporation of the organic solvent results in the creation of microcarriers [150].
  2. Spray drying can be used to produce solid microcarriers from a solution or suspension of the microcarrier material [148].
  3. EHD techniques such as electrospinning and electro-spraying can create microcarriers with precise sizes and morphologies. Using a template, it is possible to generate microcarriers with precise shapes and porosity architectures [150].
  4. Microfluidics: With fine control over size and form, microfluidic devices can create monodisperse microcarriers [151].
4. Surface Functionalization: Surface functionalization enables extra capabilities, including increased targeting, improved biocompatibility, or sustained payload release. There are many methods for surface modification, including coating with biomolecules, ligand conjugation, and attaching targeted moieties [5, 87].
5. Drug Loading: A suitable loading method should be used if the microcarriers are meant to transport a payload, such as medications or nanoparticles. Based on the payload and the microcarrier, this may include simple adsorption, encapsulation during synthesis, or surface conjugation [153].
6. Characterization of the fabricated nanoparticles and microcarrier characteristics: To evaluate their shape, content, and functional groups, methods like scanning electron microscopy (SEM), transmission electron microscopy (TEM), X-ray diffraction (XRD), and Fourier transform infrared spectroscopy (FTIR) can prove useful [153].

If drug distribution is the goal, packing the required drug into the microcarriers and encapsulating it is recommended. To understand the effectiveness of the delivery mechanism, drug release kinetics should be conducted. After that, research on toxicity and biocompatibility should be conducted. If targeted drug delivery is the goal, looking into various targeting techniques to increase the system's ability to deliver the medication precisely to the intended location is required. Finally, planning should be done for testing, validation, optimization, and continual improvement [154].

## Challenges

### Optimization of microcarrier design for improved efficacy

Even with the promising potentials of selenium and gold-infused polymer-based microcarriers, several issues must be properly resolved to increase their effectiveness for treatment, guarantee effective tumor targeting and permeation, regulate the delivery of drugs and guarantee their practical use clinically; these involve enhancing the physicochemical characteristics of microcarriers, such as size, coating charge, and discharge rates [5, 87, 91, 150, 151].

### Synergistic approaches and combination therapies

**Fig. 11:**
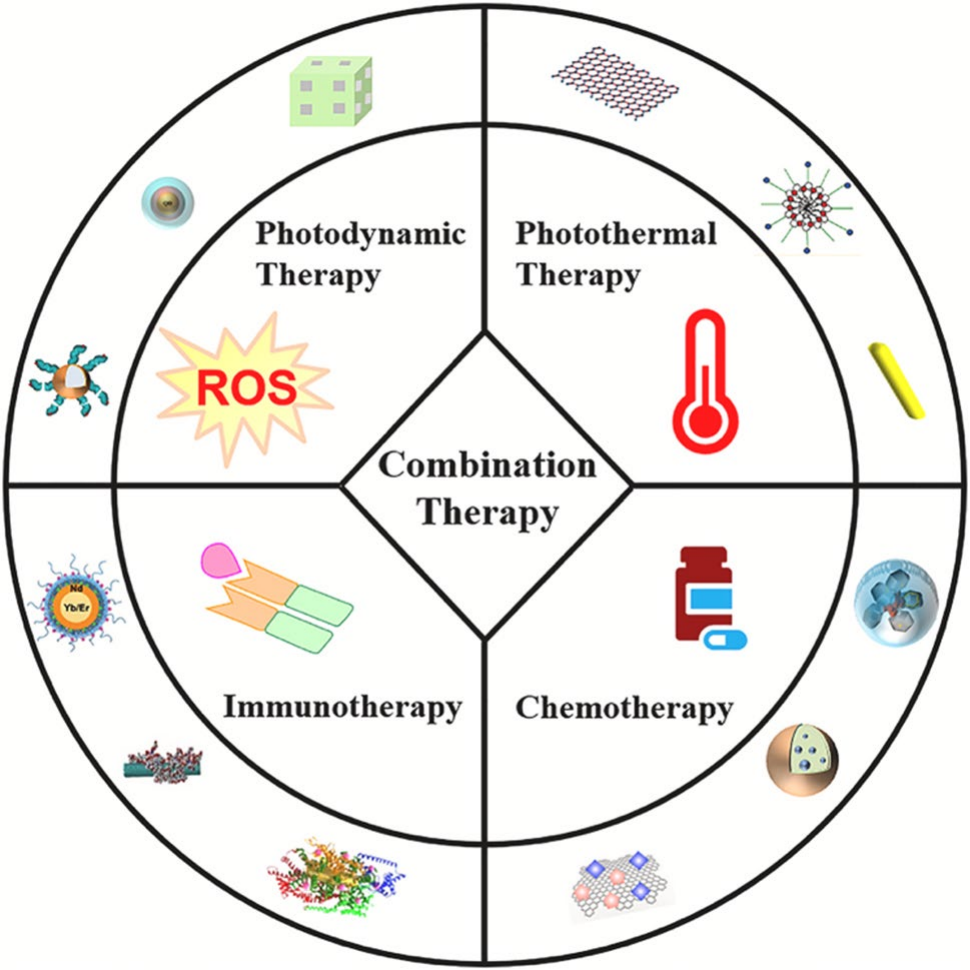
Combination therapies and synergistic approaches. Adapted from [147]

To overcome drug resistance and enhance the results of therapy, combinatorial therapy and complementary techniques must be developed. To get more benefits and personalized treatment plans, more research should be done on how to combine chemotherapy, immunotherapy, targeted therapy, and microcarriers with selenium and gold in a combinatorial approach [123, 143, 147]. Introduction of AI and machine learning aproaches synergistically with the above approaches at every step as earlier discussed will automate labourious processes, cut out trial methods and generate new insights. Figure 11 shows combination therapies and synergistic approaches possible.

### Translational potential and clinical applications

The clinical uses and applied benefits of delivery systems developed from polymers with SeNPs and AuNPs for prostate cancer treatment are encouraging [90, 115, 121]. To confirm their safety, effectiveness, and results over time, additional preclinical research and human studies is still needed. To make the transition of the technology in question into clinical practice easier, other factors, including expanding, affordability, and aspects of regulation, must be accounted for.

## Conclusion

Selenium and gold nanoparticles used in polymer-derived delivery design offer a novel and promising approach for ROS-inducing synergized photothermal treatment in prostate cancer. The synergistic effects of ROS generation and photothermal therapy provide a powerful strategy for tumor regression and improved treatment outcomes. However, further research is needed to optimize microcarrier design, explore combination therapies, and evaluate their translational potential. With continued advancements in this field, selenium and gold-incorporated microcarriers have the potential to revolutionize prostate cancer treatment by providing targeted and effective therapeutic options.

## Data Availability

Not applicable.

## Abbreviations

PTT: Photothermal therapy
PDT: Photodynamic therapy
CT: Chemotherapy
CT: Computed tomography
NP: Nanoparticle
AuNP: Gold nanoparticle
SeNP: Selenium nanoparticle
ROS: Reactive oxygen species
AuNRs: Au nanorods
AuNSs: Au nanoshells
HAuNP: Hollow Au nanoparticles
AuNCs: Au nanocluster
AuNPrs: Au nanoprisms
PTA: Photothermal agent
NIR: Near infrared radiation
EPR: Enhanced permeation and retention
PIN: Prostatic intraepithelial neoplasm
CaP: Prostate cancer cell line
